# A prospective, multicenter, observational study of long-term decitabine treatment in patients with myelodysplastic syndrome

**DOI:** 10.18632/oncotarget.6242

**Published:** 2015-10-26

**Authors:** Seong Hyun Jeong, Yoo-Jin Kim, Je-Hwan Lee, Yeo-Kyeoung Kim, Soo Jeong Kim, Sung Kyu Park, Young Rok Do, Inho Kim, Yeung-Chul Mun, Hoon Gu Kim, Won Sik Lee, Hyeon Gyu Yi, Young-Don Joo, Chul Won Choi, Suk Ran Kim, Sang Min Na, Jun Ho Jang

**Affiliations:** ^1^ Department of Hematology-Oncology, Ajou University School of Medicine, Suwon, South Korea; ^2^ Department of Hematology, Seoul St. Mary's Hospital, College of Medicine, The Catholic University of Korea, Seoul, Korea; ^3^ Department of Hematology, Asan Medical Center, Seoul, Korea; ^4^ Department of Hematology, Chonnam National University Hwasun Hospital, Jeollanam-do, South Korea; ^5^ Division of Hematology, Department of Internal Medicine, Severance Hospital, Yonsei University College of Medicine, Seoul, Korea; ^6^ Department of Internal Medicine, Soonchunhyang University Hospital, Bucheon, South Korea; ^7^ Division of Hematology-Oncology, School of Medicine, Keimyung University, Daegu, South Korea; ^8^ Department of Internal Medicine, Seoul National University, Seoul, Korea; ^9^ Department of Hematology and Oncology, School of Medicine, Ewha Womans University, Seoul, Korea; ^10^ Division of Hematology-Oncology, Department of Internal Medicine, Gyeongnam Regional Cancer Center, Institute of Health Sciences, Gyeongsang National University School of Medicine, Jinju, South Korea; ^11^ Department of Internal Medicine, Inje University College of Medicine, Inje University Busan Paik Hospital, Busan, South Korea; ^12^ Department of Internal Medicine, Inha University College of Medicine, Incheon, South Korea; ^13^ Hematology-Oncology, Department of Internal Medicine, Inje University College of Medicine, Haeundae Paik Hospital, Busan, South Korea; ^14^ Division of Oncology and Hematology, Department of Internal Medicine, Korea University Medical Center, Seoul, Korea; ^15^ Janssen Korea Medical Affairs, Seoul, Korea; ^16^ Division of Hematology and Oncology, Samsung Medical Center, Sungkyunkwan University School of Medicine, Seoul, Korea

**Keywords:** decitabine, long-term treatment, myelodysplastic syndrome

## Abstract

This prospective observational study evaluated the efficacy and safety of long-term decitabine treatment in patients with myelodysplastic syndrome (MDS). Decitabine 20 mg/m^2^/day was administered intravenously for 5 consecutive days every 4 weeks to MDS patients in intermediate-1 or higher International Prognostic Scoring System (IPSS) risk categories. Active antimicrobial prophylaxis was given to prevent infectious complications. Overall response rate (ORR), overall survival (OS), progression-free survival (PFS), and time to response were evaluated, as were adverse events. The final analysis included 132 patients. IPSS risk was intermediate-2/high in 34.9% patients. The patients received a median of 5 cycles, with responders receiving a median of 8 cycles (range, 2-30). ORR was 62.9% (complete response [CR], 36; partial response [PR], 3; marrow complete response [mCR], 19; and hematologic improvement, 25). Among responders, 39% showed first response at cycle 3 or later. OS at 2 years was 60.9%, with 17% progressing to acute myeloid leukemia. PFS at 2 years was 51.0%. Patients achieving mCR showed comparable survival outcomes to those with CR/PR. With active antibiotic prophylaxis, febrile neutropenia events occurred in 61 of 1,033 (6%) cycles. Long-term decitabine treatment with antibiotic prophylaxis showed favorable outcomes in MDS patients, and mCR predicted favorable survival outcomes.

## INTRODUCTION

Myelodysplastic syndrome (MDS) is a group of bone marrow disorders manifesting as cytopenias resulting from ineffective hematopoiesis and progressively evolving to acute myeloid leukemia (AML). Supportive treatment has long been the mainstay of treatment for a majority of patients, with allogeneic stem cell transplantation performed when available. DNA hypermethylation leading to inactivation of tumor-suppressor genes is the main pathobiologic mechanism in MDS [1-3], and the introduction of demethylating agents has changed the treatment paradigm of MDS.

Decitabine (5-aza-2′-deoxycytidine) reverses aberrant DNA hypermethylation of CpG islands by inhibiting DNA methyltransferase, resulting in reactivation of previously silenced tumor-suppressor genes [4]. Although decitabine as single-agent therapy failed to demonstrate overall survival (OS) benefit in contrast to azacitidine [5, 6], the United States Food and Drug Administration (US FDA) approved decitabine for the treatment of MDS, considering its comparable efficacy to azacitidine. Decitabine therapy has shown favorable treatment outcomes, with overall improvement rates of 42%-73% in previous studies [7-10]. Longer treatment duration may lead to better survival outcomes. A recent analysis from the AZA 001 trial showed that even patients with stable disease (SD) retained a survival benefit with azacitidine treatment and that late response could occur in one-third of patients with SD in earlier cycles [11]. Thus, continuation of treatment until disease progression is generally recommended. In previous studies, frequent, early treatment discontinuations, mainly due to febrile neutropenia and cytopenia-related infections, may have prevented patients from achieving the best clinical benefit of demethylating agents. A retrospective study showed a significant decrease in febrile episodes in patients receiving antibiotic prophylaxis [12]. We performed a multicenter, prospective observational study to determine whether long-term decitabine treatment with antibiotic prophylaxis and proper dose/schedule modification yielded better clinical outcomes in patients with MDS.

## PATIENTS AND METHODS

### Patients

The study included adult patients (≥20 years) with MDS of any WHO subtype or chronic myelomonocytic leukemia, an IPSS score of 0.5 or more, and naïve to treatment with demethylating agents. Patients were excluded if they were allergic to decitabine, were pregnant or lactating, had progressed to AML (≥20% blasts), or had concurrent malignancy. Patients with active viral or bacterial infections were not included until complete recovery. This study was reviewed and approved by the institutional review board of each participating center, and all patients provided written informed consent. The study was registered at www.clinicaltrials.gov (NCT01400633).

### Treatments

Decitabine was administered according to the 5-day intravenous outpatient schedule used in the ADOPT trial [8]. Patients received 5 consecutive injections of decitabine 20 mg/m^2^/day over 1 hour every 4 weeks. Dose modification and delay of cycles were allowed per protocol guidelines when patients experienced severe adverse effects. Subsequent cycles could be delivered if the absolute neutrophil count was >500/mm^3^ and platelet count was >30,000/mm^3^. Treatment was delayed until recovery from non-hematological toxicities, such as serum creatinine >2 mg/dL, serum glutamate-pyruvate transaminase or total bilirubin >2× upper normal limit, or active or uncontrolled infections. Active antibiotic prophylaxis was recommended to prevent infectious complications, especially in the first 4 cycles. Supportive measures, including antiemetics, transfusion, and growth factors, were allowed if needed. Continuation of decitabine treatment was recommended for at least 4 cycles, until progression or unacceptable AEs occurred.

### Study end points

The primary end point of this study was the ORR, calculated as the sum of CR, PR, mCR, and HI. Secondary end points were OS, PFS, and time to response. Patients were followed-up for survival every 12 weeks until death. Response was evaluated by central review according to the modified IWG 2006 response criteria [14]. Treatment response was evaluated after completion of each cycle. Bone marrow biopsy was recommended at every 2 cycles, at the end of treatment, and when disease progression or recurrence was suspected.

### Safety evaluation

Physical examination, complete blood count, and chemistry were performed at baseline and the first day of each cycle before initiation of treatment. The end of treatment evaluation was conducted 56 ± 7 days after the last dose of decitabine. AEs were evaluated based on the National Cancer Institute Common Terminology Criteria for Adverse Events (NCI CTCAE) ver. 4.0.

### Statistical analysis

We analyzed the ORR, OS, PFS, and time to response in the FAS population, which comprised patients who met all of the inclusion/exclusion criteria, had a baseline assessment, received at least 2 cycles of decitabine treatment, and had at least one valid post-baseline clinical response assessment. ORR was defined as the proportion of patients with CR, mCR, PR, and HI as the best response based on central review employing the IWG 2006 response criteria Confidence intervals for ORR were estimated with the Clopper-Pearson formula. OS was defined as the time from the start of decitabine treatment to death; data on survivors were censored at the last follow-up. PFS was defined as the time from the date of first decitabine administration to progression, relapse after showing CR or PR, or death, whichever occurred first. Survival was calculated using the Kaplan-Meier method, and log-rank tests were used to compare the OS distribution by variables (best treatment response type and achievement of mCR). Time to first response and time to best response were analyzed with frequency and descriptive statistics (median, min-max) in each treatment cycle. Logistic regression analysis was used to assess the efficacy of decitabine post hoc within subgroups defined by the following baseline characteristics, which were determined to have effects on ORR: age (≥65, <65), gender, period of MDS (≥ 1 year, <1 year), WHO subtype, French-American-British (FAB) subtype, IPSS category, and karyotype. Safety was analyzed in all patients who received at least one dose of decitabine. All *P*-values were two-sided and statistical significance was accepted at the level of *P*<0.05.

## RESULTS

### Patient characteristics

**Figure 1:**
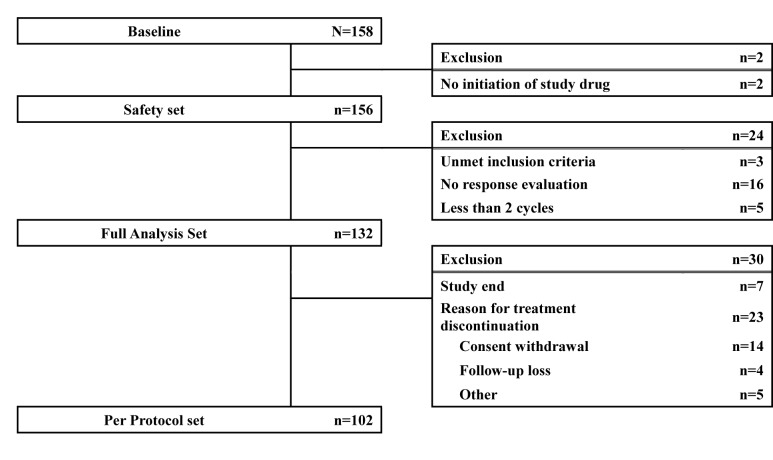
Patient flowchart

The study was conducted at 33 centers in Korea from December 2010 to October 2011. Of the 158 patients enrolled, 2 patients were excluded from the safety analysis for non-initiation of the study drug (decitabine), and 24 patients were excluded from the full analysis set (FAS) for not meeting inclusion criteria (3 patients), dropout before 2 cycles (5 patients), and not undergoing response evaluation (16 patients). Thus, 132 patients were included in the FAS (Figure 1). Their median age was 63 years (range, 20-82 years), and a majority of the patients were male (57.6%). The median duration of disease was 19 days (range, 1-3,011 days). Patients were categorized according to the International Prognostic Scoring System (IPSS) into intermediate-1 (Int-1; 65%), intermediate-2 (Int-2; 27%), and high (7.6%) risk groups. Five patients had secondary MDS. At the baseline evaluation, karyotype was good in 75 patients (57%), intermediate in 27 (20%), and poor in 22 (17%); karyotyping data was unavailable in 8 patients. Only 3 patients had previously received other treatments before decitabine treatment (androgen, 2; erythropoietin, 1). Patient characteristics are summarized in Table 1.

**Table 1:** Patient characteristics (N=132)

Characteristics	n (%)
Gender	
Male	76 (57.58%)
Female	56 (42.42%)
Age (years)	
Median (range)	63 (20-82)
Duration of disease (days)	
Median (range)	19 (1-3,011)
Comorbidity	
Yes	38 (28.79%)
No	94 (71.21%)
MDS type	
De novo	127 (96.21%)
Secondary	5 (3.79%)
WHO subtype	
RCUD	4 (3.03%)
RARS	3 (2.27%)
RCMD	40 (30.30%)
RAEB-1	30 (22.73%)
RAEB-2	36 (27.27%)
MDS-U	6 (4.55%)
Del(5q)	1 (0.76%)
CMML-1	7 (5.30%)
CMML-2	4 (3.03%)
Unclassified	1 (0.76%)
IPSS risk category	
Intermediate-1	86 (65.15%)
Intermediate-2	36 (27.27%)
High	10 (7.58%)
ECOG performance status	
0, 1	109 (82.58%)
≥2	23 (17.42%)
Karyotype at baseline	
Good	75 (56.82%)
Normal	69 (52.27%)
Other	6 (4.55%)
Intermediate	27 (20.45%)
Poor	22 (16.67%)
Not done	8 (6.06%)

CMML, chronic myelomonocytic leukemia; Del, deletion; ECOG, Eastern Cooperative Oncology Group; IPSS, International Prognostic Scoring System; MDS, myelodysplastic syndrome; MDS-U, unclassifiable MDS; RAEB, refractory anemia with excess of blasts; RARS, refractory anemia with ringed sideroblasts; RCMD, refractory cytopenia with multilineage dysplasia; RCUD, refractory cytopenia with unilineage dysplasia; WHO, World Health Organization

**Table 2:** Treatment response

Response	n (%)	
CR	36	(27.27%)
PR	3	(2.27%)
mCR with HI	8	(6.06%)
mCR without HI	11	(8.33%)
HI only*	25	(18.94%)
Patient experienced HI	72	
SD^#^	48	(36.36%)
ORR (CR + PR + mCR + HI)	83	(62.88%)
95% CI^+^	(54.04%	−71.12%)
CR + PR + mCR + HI + SD	131	(99.24%)
Failure	1	(0.76%)
Cytogenetic response	55**	
CR	14	(25.45%)
PR	5	(9.09%)
NE/ND	36	(65.45%)

CR, complete response; HI, hematologic improvement; HI-E, erythroid response; HI-N, neutrophil response; HI-P, platelet response; IWG, International working group; mCR, marrow complete response; ND, not done; NE, not evaluable; ORR, overall response rate; PR, partial response; SD, stable disease

* HI only: HI in SD and NE/ND

# SD excluding other than HI

+ 95% confidence interval for proportion of ORR

** In case of abnormal Karyotype (Good-Other, Intermediate, Poor)

### Treatment exposure and response

A total of 1,033 cycles were delivered to 132 patients (median, 5 cycles), with 84% patients receiving 3 or more cycles and 47% receiving 6 or more cycles. The median number of cycles among responders was 8 (range, 2-30 cycles). The mean interval between cycles was 36.8 ± 9.4 days. Dose modification was required in 8 patients. At the time of analysis, 7 patients remained adherent to the treatment. Reasons for treatment discontinuation included failure to achieve response (11%), progression (8%), adverse events (AEs; 7%), proceeding to hematopoietic stem cell transplantation (HSCT; 26%), progression to AML (13%), progression after achieving response (5%), patient decision (11% ), death (12%), and loss to follow-up loss or other reasons (7%). Prophylactic antimicrobial agents were administered to 86% patients, including antibacterials (aminopenicillin or quinolone) to 83.3%, antifungals (fluconazole or itraconazole) to 77%, and antivirals (acyclovir) to 28%.

**Figure 2:**
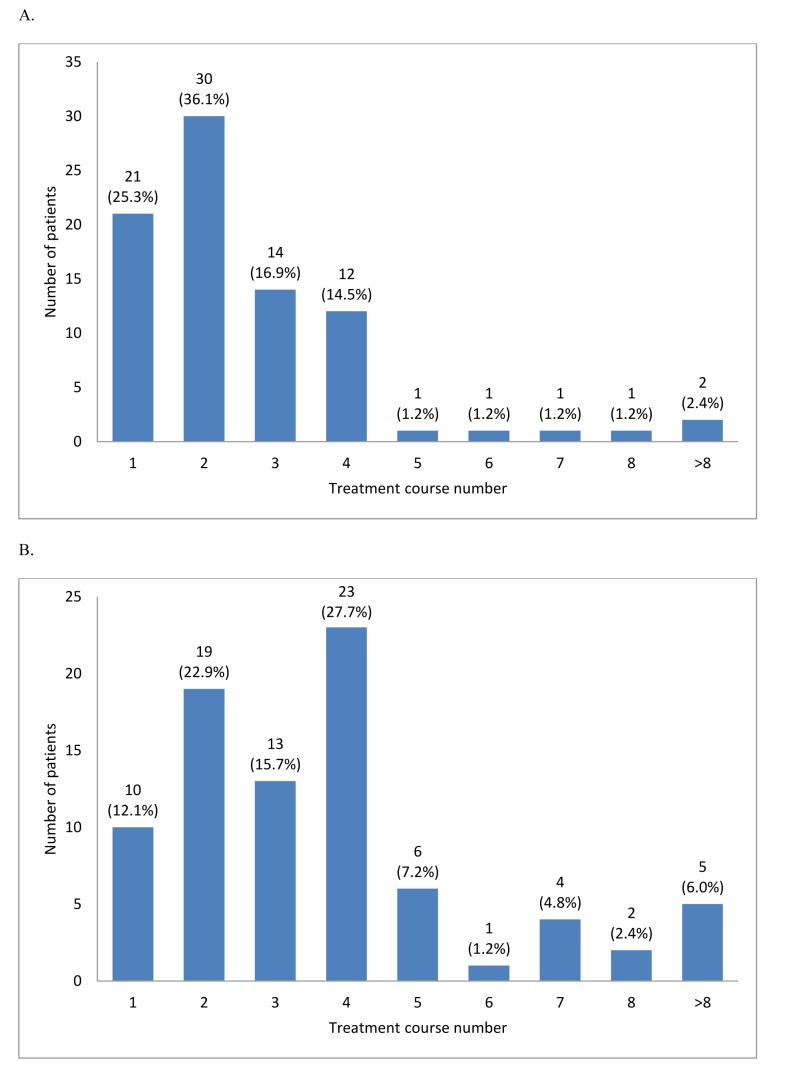
Time to first response A. and time to best response B

The overall response rate (ORR) was 62.9% (95% CI, 54.0%-71.1%): complete response (CR), 27.3%; partial response (PR), 2.3%; marrow complete response (mCR), 14.4%; and hematologic improvement (HI), 18.9%. Initial response was achieved within 2 cycles in 61% patients; however, 39% patients showed initial response at cycle 3 or later (31% at cycles 3 and 4; Figure 2A). The best response peaked at cycle 4 (28%; Figure 2B). HI was observed in 44.4% patients (erythroid response [HI-E], 26.2%; platelet response [HI-P], 31%; and neutrophil response [HI-N], 16.7%). The median number of cycles to HI was 2 (range, 1-13). Cytogenetic response was noted in 19 of 55 patients (34.6%; CR, 25.5% and PR, 9.1%) with abnormalities at baseline. Progression to AML was 10.8% at 1 year and 17% at 2 years. Karyotype at diagnosis was a significant prognostic factor for ORR (Table 3). Patients with good or poor karyotype showed better ORR than those with an intermediate type.

**Table 3:** Prognostic factor analysis for overall response

Characteristic	Response		*P*-value‡	Odds ratio	95% CI		*P*-value*
	n/N	(%)			Lower	Upper	
**Age, years**							0.7925
**≤65**	46/72	(63.89%)	0.7924	1			
**>65**	37/60	(61.67%)		0.909	0.448	1.847	
**Sex**							0.7740
**Male**	47/76	(61.84%)	0.774	1			
**Female**	36/56	(64.29%)		1.111	0.543	2.273	
**Duration of MDS**							0.0985
**≤ 1 year**	76/116	(65.52%)	0.0911	1			
**> 1 year**	7/16	(43.75%)		0.409	0.142	1.181	
**WHO subtype**							0.3437
**RCUD**	1/4	(25.00%)	0.2	1			
**RARS**	2/3	(66.67%)		6.000	0.221	162.531	
**RCMD**	22/40	(55.00%)		3.667	0.351	38.345	
**RAEB-1**	23/30	(76.67%)		9.857	0.880	110.425	
**RAEB-2**	26/36	(72.22%)		7.800	0.723	84.091	
**MDS-U**	2/6	(33.33%)		1.500	0.089	25.392	
**Del(5q)**	1/1	(100.00%)		>999.999	<0.001	>999.999	
**CMML-1**	3/7	(42.86%)		2.250	0.149	33.933	
**CMML-2**	2/2	(50.00%)		3.000	0.150	59.890	
**Unclassified**	1/1	(100.00%)		>999.999	<0.001	>999.999	
**IPSS risk category**							0.5265
**Intermediate-1**	53/86	(61.63%)	0.5062	1			
**Intermediate-2**	22/36	(61.11%)		0.978	0.440	2.175	
**High**	8/10	(80.00%)		2.491	0.498	12.451	
**Karyotype at baseline**							0.0239
**Good (normal or other)**	53/75	(70.67%)	0.0191	1			
**Intermediate**	11/27	(40.74%)		0.285	0.114	0.712	0.0112**
**Poor**	15/22	(68.18%)		0.890	0.319	2.481	0.3211**

CMML, chronic myelomonocytic leukemia; CR, complete response; Del, deletion; HI, hematologic improvement; IPSS, International Prognostic Scoring System; mCR, marrow complete response; MDS, myelodysplastic syndrome; MDS-U, unclassifiable MDS; ORR, overall response rate; PR, partial response; RAEB, refractory anemia with excess of blasts; RARS, refractory anemia with ringed sideroblasts; RCMD, refractory cytopenia with multilineage dysplasia; RCUD, refractory cytopenia with unilineage dysplasia

* *P*-value is obtained by logistic regression (Wald test – testing global null hypothesis)

** *P*-value is obtained by logistic regression

‡ *P*-value is obtained by Chi-square test

### Survival data

The median follow-up duration was 9.5 months. Among 132 patients in FAS, fifty-nine (44.7%) patients died during the treatment (16.7%) or follow-up period (28%). Causes of death were disease progression (24%), AEs (20%), and other reasons (56%). Other reasons of death included pneumonia in 10 patients and sepsis/septic shock in 6 patients.

**Figure 3:**
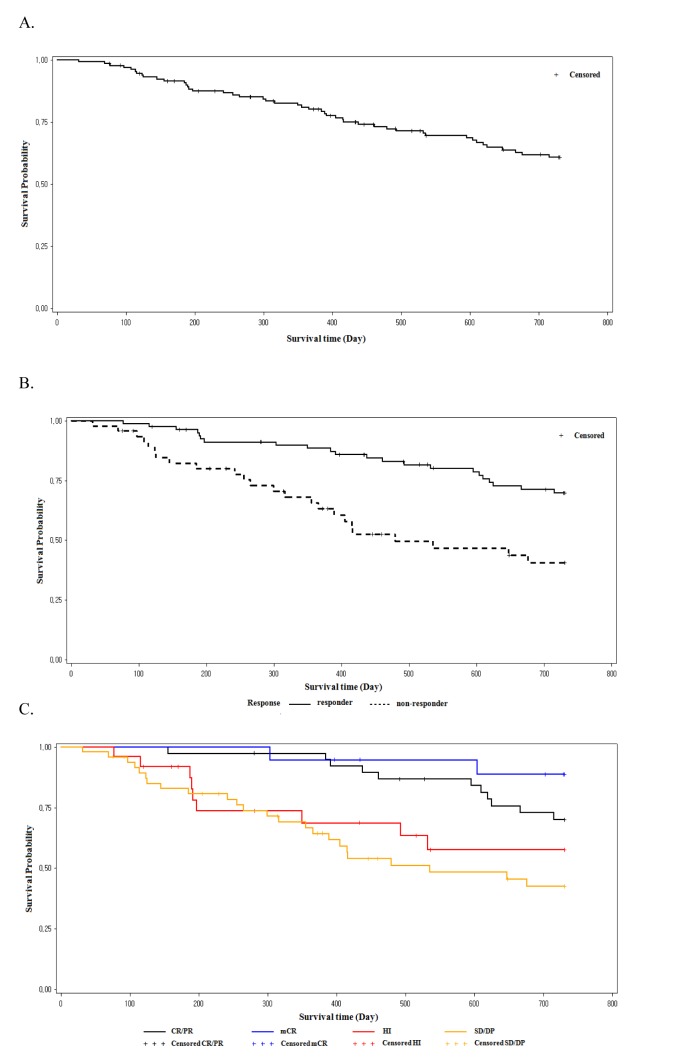
Overall survival (OS; A.), OS in responders vs. non-responders B., and OS by response type C

The 1-year and 2-year OS was 80.9% (95% CI, 72.9%-86.8%) and 60.9% (95% CI, 51.3%-69.1%), respectively. The progression free survival (PFS) rate was 70.0% (95% CI, 61.1%-77.2%) at 1 year and 51% (95% CI, 41.5%-59.6%) at 2 years. Patients who achieved response with decitabine treatment (responders) showed better OS at 1 year (88.8% vs. 66.7%, *P*=0.0035) and 2 years (70.6% vs. 42.5%, *P*=0.0014) than those who did not achieve response (nonresponders; Figure 3B). Patients who achieved mCR showed comparable survival with those achieved CR or PR, regardless of HI (Figure 3C). Patients who achieved HI showed better survival than those who did not. This was statistically significant in the higher risk IPSS groups (INT-2 and high), but not in the lower risk group.

### Adverse events

The safety analysis included 156 patients. Febrile neutropenia was the most common AE, considered to be possibly related to the study drug in 23.7% patients. Febrile neutropenia events occurred in 61 of 1,033 (6%) cycles in 37 patients in the FAS. Grade III or more AEs included neutropenia (21.2%), anemia (12.8%), thrombocytopenia (15.4%), and febrile neutropenia (23.7%). Non-hematologic AEs occurring in >10% patients included nausea (27.6%), decreased appetite (23.7%), and pyrexia (23.1%). Documented infection included upper respiratory tract infection in 5 patients, pneumonia in 6, sepsis in 5, and cellulitis in 2. Grade III or IV non-hematologic AEs included nausea (1.3%), decreased appetite (3.2%), and pyrexia (3.9%).

## DISCUSSION

This is one of the largest multicenter studies prospectively evaluating the efficacy of decitabine in patients with MDS. In previous studies, the primary cause of early discontinuation of decitabine treatment, other than treatment failure, was cytopenia-related infections. Therefore, to prevent early dropout, all patients in this study were to receive antibacterial prophylaxis as well as dose and schedule modification if they experienced cytopenias at the first cycle. This led to a reduction in febrile neutropenia events to 6% (62 of 1,033 cycles) in contrast to 11%-14% reported with the same treatment schedule in previous studies [8, 9]. This translated into a decrease in early discontinuation rates (84% patients received at least 3 cycles) and increase in the median number of cycles.

Overall treatment outcomes in this study compare favorably with those from previous studies using the 5-day outpatient protocol [8-10]. The CR and ORR in this study were somewhat higher than those in the DIVA study [9]. This could be attributed mainly to the patient characteristics, with our study including more IPSS Int-1 patients than previous studies. Furthermore, most patients were newly diagnosed and were treatment naive. Only 5 patients with secondary MDS were included. In addition, 47% patients received at least 6 cycles, with the responders receiving a median of 8 cycles. Baseline cytogenetic data was available for 94% patients. Of the 55 patients with cytogenetic abnormalities at baseline, 19 patients (35%) showed cytogenetic response (14 CR and 5 PR). Interestingly, the response rate to decitabine was inferior in patients with an intermediate karyotype as compared to patients with good or poor risk cytogenetics. Thus, decitabine may have better effects on clones with more cytogenetic/genetic alterations.

The clinical significance of mCR remains unclear. In previous studies, 15%-23% decitabine-treated patients showed mCR [5, 9, 10]. The 14% patients who achieved mCR in our study showed comparable survival outcomes with the CR/PR patients regardless of HI status. mCR seems to be an analogue of CR with incomplete blood count recovery (CRi) in AML, reflecting a low tumor burden but with decreased marrow function. Because normal hematopoiesis is suppressed in MDS, it is natural that a substantial proportion of patients show mCR after successful treatment with demethylating agents. In a retrospective analysis, patients with mCR showed comparable outcomes to those with CR after HSCT [13]. In the present study, patients who achieved mCR showed favorable survival outcomes regardless of HSCT status. However, this finding is discordant with a previous study, where patients achieving mCR showed inferior survival outcomes compared to those with CR or PR [9]. Further studies are required to clarify this effect.

HI was a significant predictor of OS in the DIVA study [9]. This finding was confirmed in our study. As in the DIVA study [9], HI was associated with better survival in higher risk patients (IPSS INT-2 or high), but not in lower risk patients (INT-1). Recently, an analysis of higher risk patients treated with azacitidine showed that patients who achieved HI without response (CR or PR) also experienced a survival benefit (hazard ratio 0.19; 95% CI, 0.08-0.46; *P*<0.001) [11]. Thus, HI could be accepted as a predictor of better survival in higher risk MDS patients treated with demethylating agents.

In previous studies, response to demethylating agents was observed early in the course of treatment, usually within 2 cycles [5, 6, 9, 10]. In our study, however, a higher proportion of patients (37.6%) showed first response only at cycle 3 or later. We do not have a clear explanation for this observation. However, the median cycle duration was 35 days in our study, which is significantly longer than in previous studies. The lower early dose intensity in our study than in the other studies may explain the difference. However, the ORR in our study was not inferior to those in other studies. As shown in previous studies, the number of treatment cycles is a significant factor determining the effect of demethylating agents [5, 6, 10]. Thus, prolonged administration of decitabine is required for the treatment of MDS. In addition, considering that a significant number of patients respond to decitabine only after 3 or more cycles, effectiveness of decitabine therapy should be assessed after completion of at least 4 cycles, especially in patients showing SD in earlier cycles. This finding was supported by a recent analysis of the AZA 001 trial [11].

The major difference between the AZA-001 and GMDSSG/EORTC 06011 trials was the median number of treatment cycles (9 vs. 4) [5, 6]. In addition, the M. D. Anderson Cancer Center study comparing 3 decitabine schedules showed that a high dose-intensity schedule induces more favorable responses [10]. To maximize the treatment outcomes of demethylating agents, both dose-intensity and treatment cycles are important. Thus, prevention of early drop-out and continuation of decitabine treatment until failure is recommended.

In conclusion, long-term decitabine treatment was effective for Int-1 or higher risk patients with MDS, with an acceptable toxicity profile. Appropriate antibacterial prophylaxis was effective in reducing early febrile neutropenia events. Patients with mCR showed comparable survival to those with CR/PR, and mCR can be considered a favorable response to decitabine treatment.
